# Growth of Hydrothermally Derived CdS-Based Nanostructures with Various Crystal Features and Photoactivated Properties

**DOI:** 10.1186/s11671-016-1490-x

**Published:** 2016-05-23

**Authors:** Yuan-Chang Liang, Tsai-Wen Lung

**Affiliations:** Institute of Materials Engineering, National Taiwan Ocean University, Keelung, 20224 Taiwan

**Keywords:** Crystal growth, Microstructures, Heterostructure, Photoactivity

## Abstract

CdS crystallites with rod- and flower-like architectures were synthesized using a facile hydrothermal growth method. The hexagonal crystal structure of CdS dominated the growth mechanisms of the rod- and flower-like crystallites under specific growth conditions, as indicated by structural analyses. The flower-like CdS crystallites had a higher crystal defect density and lower optical band gap value compared with the rod-like CdS crystallites. The substantial differences in microstructures and optical properties between the rod- and flower-like CdS crystallites revealed that the flower-like CdS crystallites exhibited superior photoactivity, and this performance could be further enhanced through appropriate thermal annealing in ambient air. A postannealing procedure conducted in ambient air oxidized the surfaces of the flower-like CdS crystallites and formed a CdO phase. The formation of heterointerfaces between the CdS and CdO phases mainly contributed to the improved photoactivity of the synthesized flower-like CdS crystallites.

## Background

Semiconductor photocatalysts have attracted considerable attention because of their successful utilization of solar energy for solving environmental remediation problems. However, because of their wide band gap, most photocatalysts are active only under ultraviolet light, which occupies several percentages of the received solar energy [1, 2]. The development of efficient photocatalysts for visible light irradiation has therefore become a critical scientific challenge for the photocatalyst community [3–5]. Compared with the abundant wide band gap semiconductor photocatalysts, photocatalysts with a visible-light band gap are relatively limited in number. Among the reported candidates of semiconductors with a visible-light band gap, CdS is a vital member of the II–VI semiconductor group with a direct band gap of approximately 2.4 eV, and it has high refraction index, excellent transport properties, and suitable energetics for harvesting solar light for photoactivated device applications. Several studies have fabricated CdS photocatalysts with various morphologies and scales for photoactivated device applications [5–8]. Among various synthesis methods, the hydrothermal synthesis route has been considered one of the most promising synthetic routes for fabricating binary semiconductor crystals because of its low process cost, easy process control, and high product reproducibility [9].

The photocatalytic activity of semiconductors is highly associated with their microstructures, which are substantially dominated by the crystal growth mechanisms under specific growth conditions. The crystal morphology, size, and composition are major factors influencing the efficiency of light absorption and photocatalytic performance [10–12]. Therefore, understanding the correlation between the crystal growth mechanism and the crystal feature is crucial in designing semiconductor photocatalysts with high photocatalytic performance. Recently, semiconductor heterostructures comprising two compounds have attracted research attention and have been used to improve photoactivated properties compared with the corresponding individual constituents [10, 13–15]. A suitable band alignment between the constituents is an effective method for separating photoinduced electron–hole pairs and enhancing photocatalytic performance [14, 16, 17]. Preparing two semiconductor compounds for heterostructures usually requires a complex and incompatible two-step process, and thus hinders the actual applications of such semiconductor heterostructures in photodegradation. Therefore, the selection of a simple synthesis process and constituent compound for a CdS-based heterostructure is crucial to realize its photocatalytic applications. CdO is an n-type semiconductor with a band gap in the visible-light region [18, 19]. Incorporating CdO into CdS to form a heterostructure might be favorable for improving the photoactivated properties of CdS-based materials because of the special band alignment structure between CdO and CdS [20]. The current study investigated the crystal growth, crystal features, and optical properties of rod- and flower-like CdS crystallites synthesized using a facile hydrothermal method. Moreover, a simple postannealing procedure was adopted to fabricate the CdS–CdO heterostructure. The photocatalytic performance levels of the CdS crystallites with various morphologies and CdO to form a heterostructure were compared and discussed on the basis of the differences in their microstructures.

## Methods

In this study, CdS crystallites with various morphologies were synthesized using a hydrothermal method. Ethylenediamine solution was used as a surfactant to hydrothermally synthesize high-density rod-like CdS crystallites. Moreover, flower-like CdS crystallites were synthesized without ethylenediamine-assisted growth. The precursor solution for the hydrothermal synthesis comprised cadmium nitrate and thiourea with a molar ratio of 1:5 and was balanced with ethylenediamine solution or deionized water. The precursor solution was stirred for 10 min and then transferred into a Teflon autoclave to undergo a hydrothermal synthesis reaction. The temperature for the hydrothermal synthesis reactions was fixed at 170 °C and maintained for different durations. Finally, the reaction system was cooled to room temperature naturally, and the final precipitates were then washed in deionized water and dried in an oven. The CdO@CdS heterostructure was prepared by subjecting the flower-like CdS crystallites to thermal postannealing at 400 °C for 5 min in ambient air.

Sample crystal structures were investigated by X-ray diffraction (XRD; Bruker D2 PHASER) using Cu Kα radiation. The morphologies of the as-synthesized samples were characterized by scanning electron microscopy (SEM; Hitachi S-4800), and high-resolution transmission electron microscopy (HRTEM; Philips Tecnai F20 G2) was used to investigate the detailed microstructures of the samples. Room-temperature-dependent photoluminescence (PL; HORIBA HR800) spectra were obtained using the 325-nm line of a He–Cd laser. The diffuse reflectance spectra of the samples were recorded by using UV–Vis spectrophotometer (Jasco V750). Photocatalytic activity of as-prepared samples were performed by comparing the degradation of 10^−6^ M aqueous solution of methylene blue (MB) with various CdS samples as catalysts under visible light irradiation (*λ* > 420 nm). For each photodegradation test, 50 mg of CdS catalysts powdered sample was dispersed in 20 mL of MB solution. After the photodegradation reaction, the supernatant solution was measured by UV–Vis spectrophotometer in the wavelength range of 400–700 nm and analyzed the photodegradation ratio at the maximum absorption wavelength (~663 nm) of MB aqueous solution. The photodegradation size is defined as (*C*/*C*_o_), where *C*_o_ is the concentration of aqueous MB without irradiation after dark adsorption equilibrium and *C* is the concentration of aqueous MB corresponding to a given visible light irradiation duration.

## Results and Discussion

**Fig. 1:**
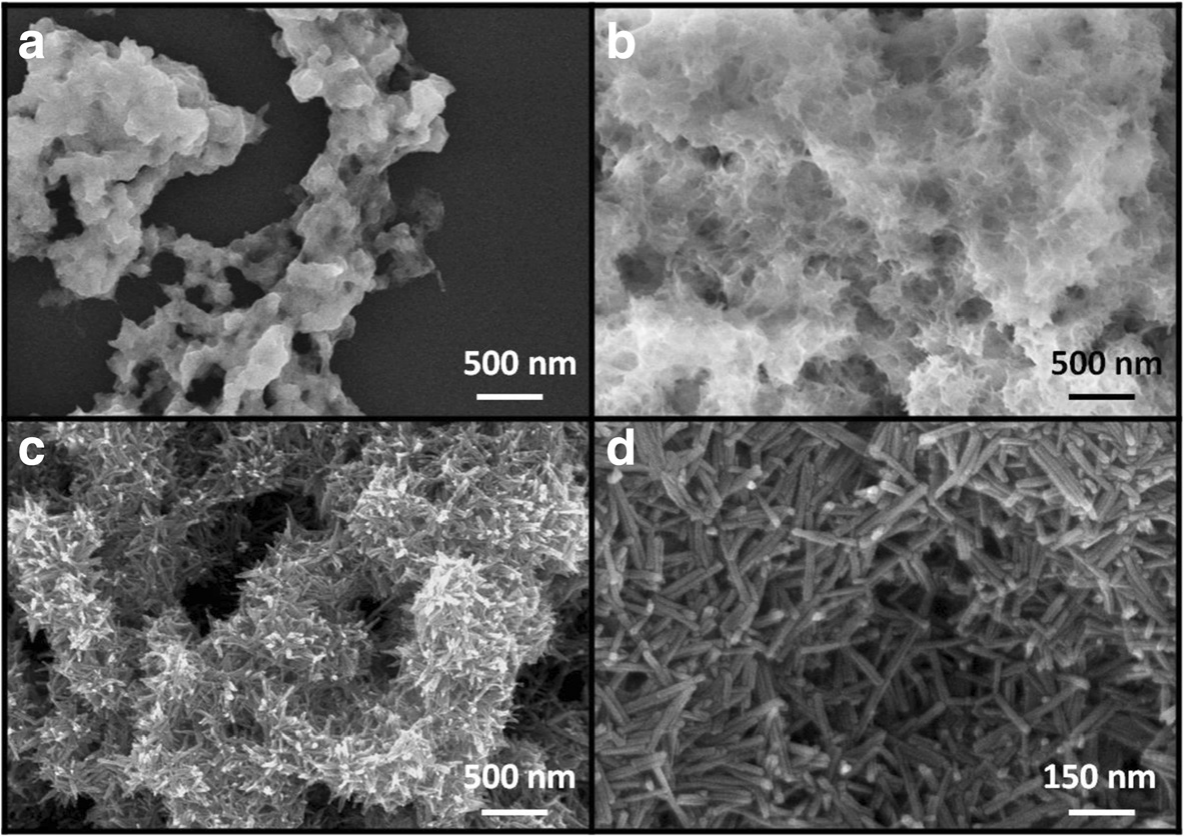
SEM micrographs of rod-like CdS crystallites hydrothermally synthesized with the following different growth durations: **a** 0.5, **b** 1, and **c** 3 h. **d** A high-magnification image of the local region in **c**

**Fig. 2:**
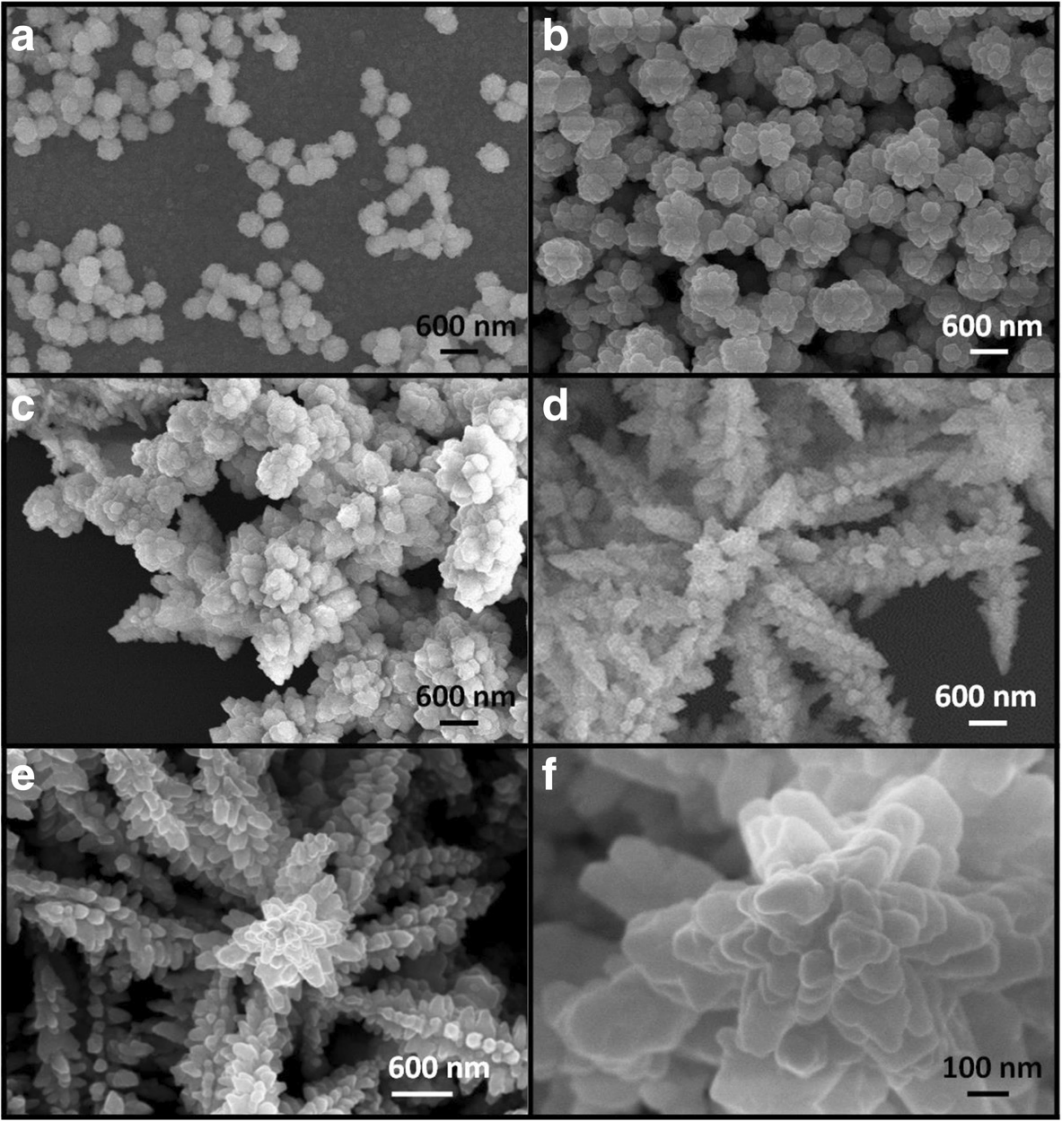
SEM micrographs of flower-like CdS crystallites hydrothermally synthesized with the following different growth durations: **a** 1, **b** 1.5, **c** 2, and **d** 5 h. **e** Image of a single flower-like CdS. **f** A high-magnification image taken from the *top region* of the flower-like CdS in **e**

Figure 1a–c depicts the SEM images of different growth stages of the rod-like CdS crystallites for various hydrothermal growth durations. Figure 1a shows that the hydrothermal precursor solution colloids have begun to form aggregates to provide nucleation sites in the initial stage of the hydrothermal growth. The surface of the aggregates turned thorny with increased hydrothermal growth duration, demonstrating that the CdS crystals have a tendency to grow along particular orientations from the surfaces of the initially formed aggregates (Fig. 1b). As the hydrothermal growth duration continued increasing, the as-formed tiny thorny crystals further grew because of the continuous supply of many CdS molecules from the precursor solution. These molecules were absorbed into the tiny thorny crystals and mainly grew along a specific crystal direction; finally, large CdS clusters with clear rod-like CdS extrusions were successfully formed on the substrate with a sufficient hydrothermal growth duration (Fig. 1c) [21]. As indicated in the high-magnification SEM image in Fig. 1d, the length of the CdS nanorods was approximately 100–250 nm, and the diameter of the CdS nanorods was approximately 10 nm. Figure 2a–d depicts the SEM images of the different growth stages of the flower-like CdS crystallites for various hydrothermal growth durations (1, 1.5, 2, and 5 h, respectively). Figure 2a shows CdS pellets with a size of approximately 150–200 nm dispersed on the substrate during the initial growth stage of the hydrothermal crystal growth process. When the hydrothermal growth duration was further increased to 1.5 h, the size of the CdS pellets increased to approximately 400–650 nm (Fig. 2b). Pellet-like crystals with a clear rugged surface were formed during this stage. The CdS crystals were coarsened by their aggregation with neighboring CdS pellets as the growth duration increased (Fig. 2c); many CdS crystal branches with lengths of several micrometers were formed because the CdS crystals continued to grow along specific orientations from various facets of the aggregated pellets for the prolonged growth duration. Finally, CdS crystals with a flower-like architecture were formed for sufficient hydrothermal growth duration (Fig. 2d). The flower-like CdS crystals comprised small secondary twigs on the main CdS branches, which were preformed from the crystal growth of coarse CdS pellets, as exhibited in Fig. 2c. The image of a single flower-like CdS and its high-magnification version obtained from the top region of the image (Fig. 2e, f) demonstrated that these secondary twigs had an angle interval of approximately 60° between each CdS twig, showing a clear extended secondary crystal growth from the hexagonal phase of main CdS branches. The lengths of the CdS branches were approximately 2.5–4 μm, and the sizes of the CdS twigs reached several hundred nanometers.

**Fig. 3:**
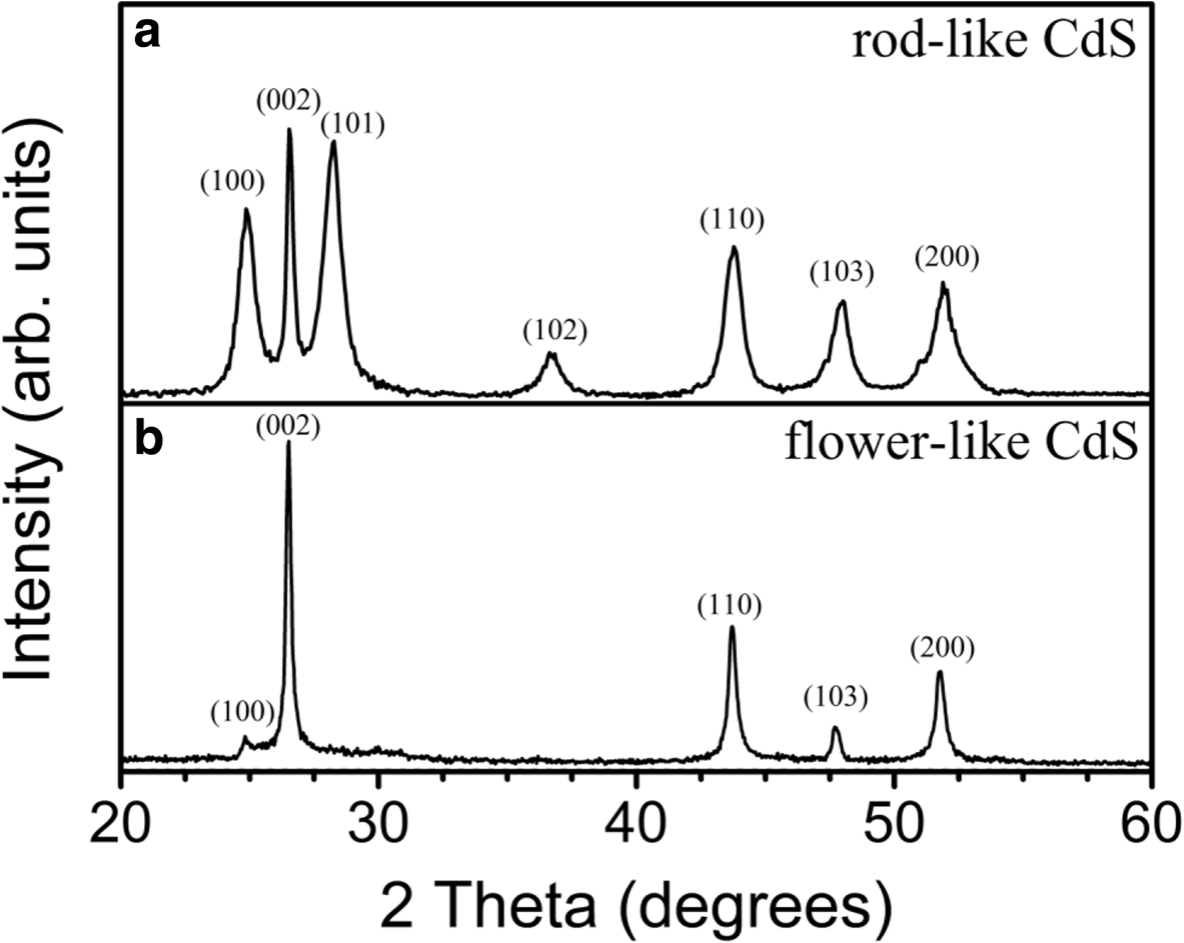
XRD patterns of CdS crystallites with various morphologies. **a** Rod-like CdS. **b** Flower-like CdS

Figure 3a, b shows the XRD patterns of the rod- and flower-like CdS crystallites, respectively. Bragg reflections were observed for the rod-like CdS crystals and were ascribed to the hexagonal phase of CdS (JCPDS no. 00-041-1049); moreover, no other impurity phases were detected. The flower-like CdS crystals also exhibited Bragg reflections originating from the hexagonal phase of CdS. Notably, the peak intensity of hexagonal CdS (002) was considerably stronger than other peaks, indicating that the flower-like CdS crystals were preferentially grown along the *c*-axis orientation of the hexagonal phase.

**Fig. 4:**
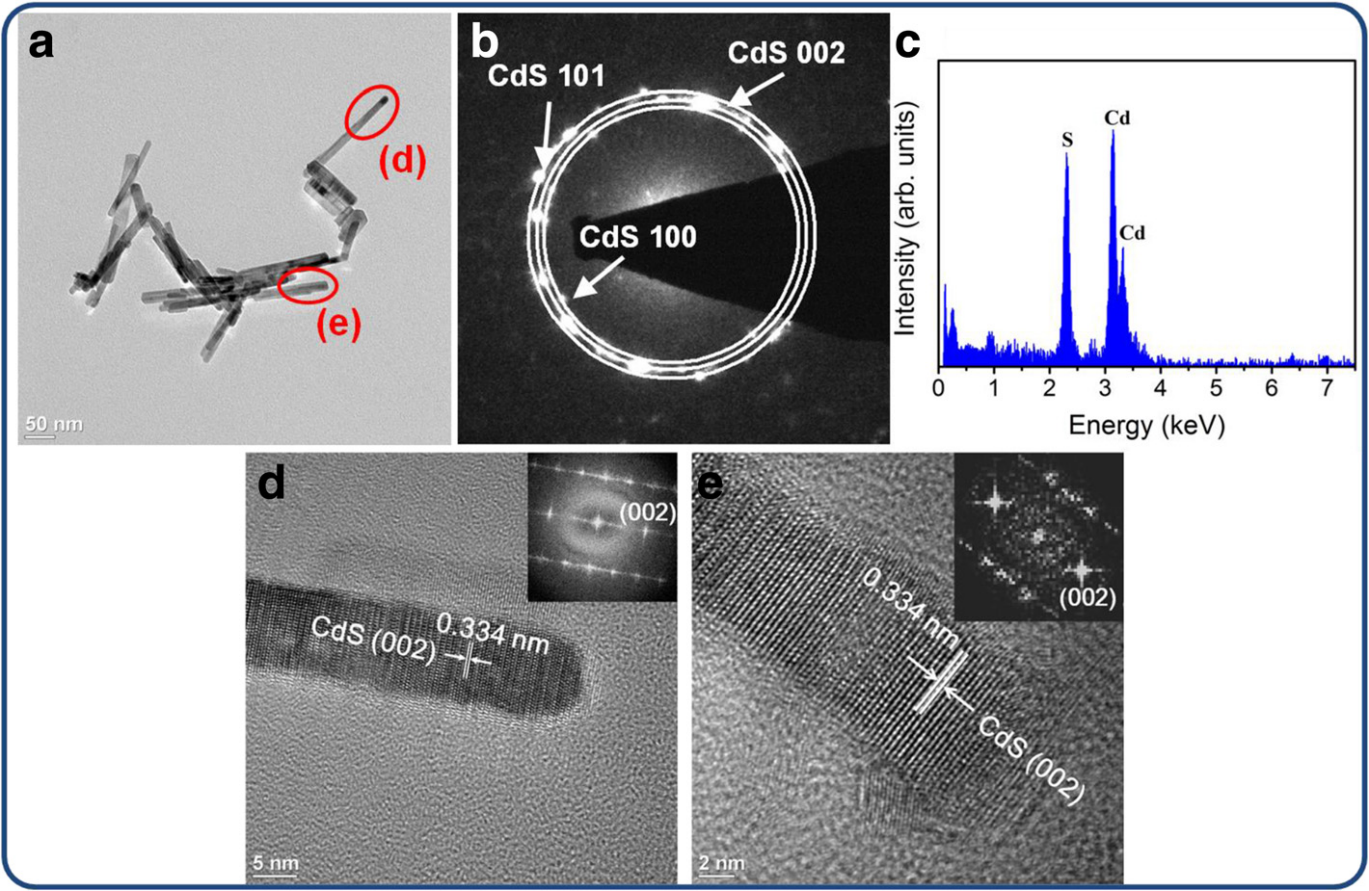
TEM analyses of rod-like CdS crystallites. **a** Low-magnification TEM image. **b** SAED pattern of grouped rod-like CdS crystallites. **c** EDS spectrum taken from the rod-like CdS crystallites. **d–e** HRTEM images taken from the edges of the rod-like CdS crystallites marked with *red circles* in **a**, and the corresponding FFT patterns are shown in the *insets*

**Fig. 5:**
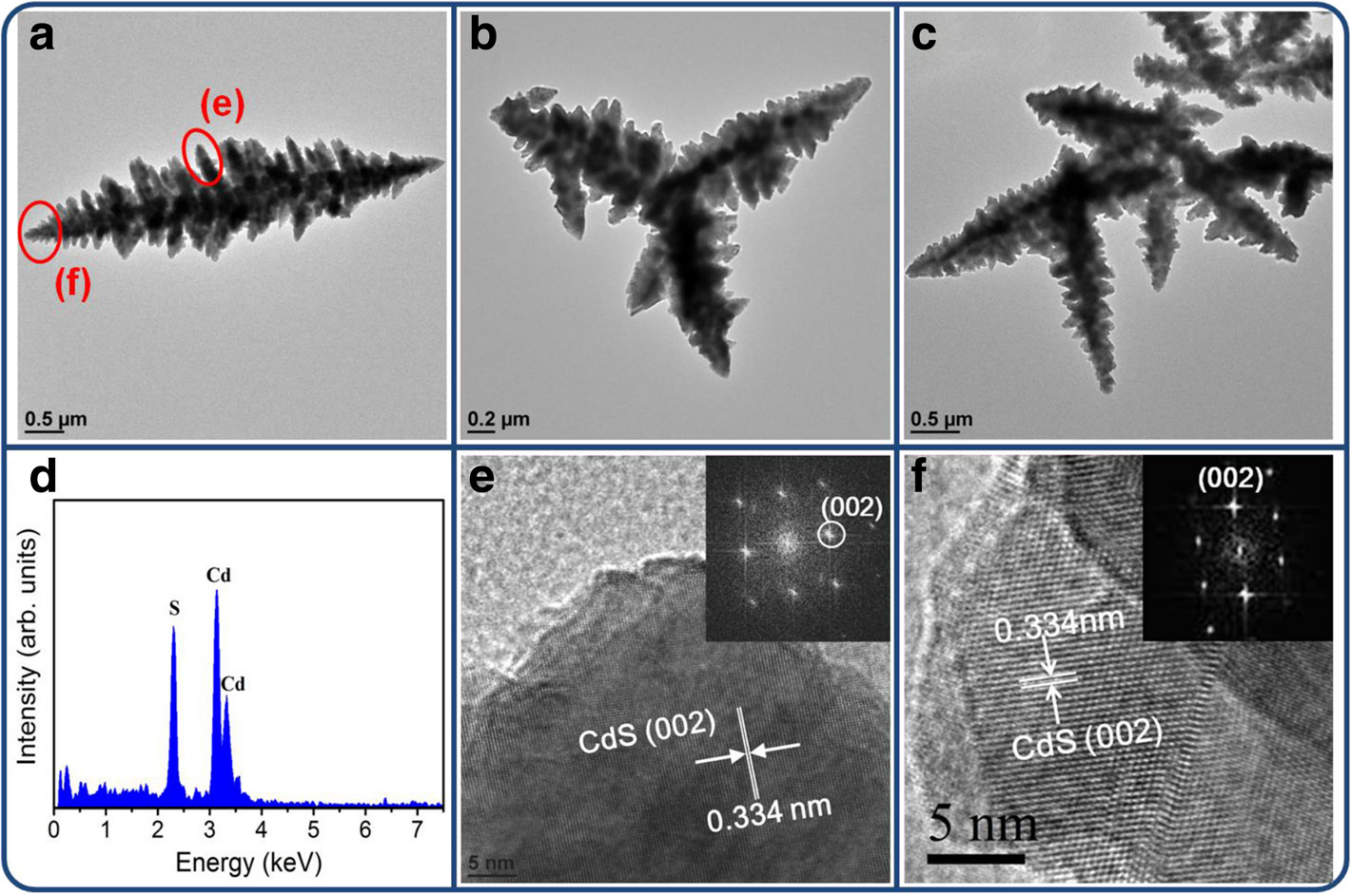
TEM analyses of flower-like CdS crystallites. **a–c** Low-magnification TEM images of single and grouped flower-like CdS crystallites. **d** EDS spectrum taken from the CdS crystallite in **a. e–f** HRTEM images taken from the edges of the flower-like CdS crystallites marked with *red circles* in **b**, and the corresponding FFT patterns are shown in the *insets*

**Fig. 6:**
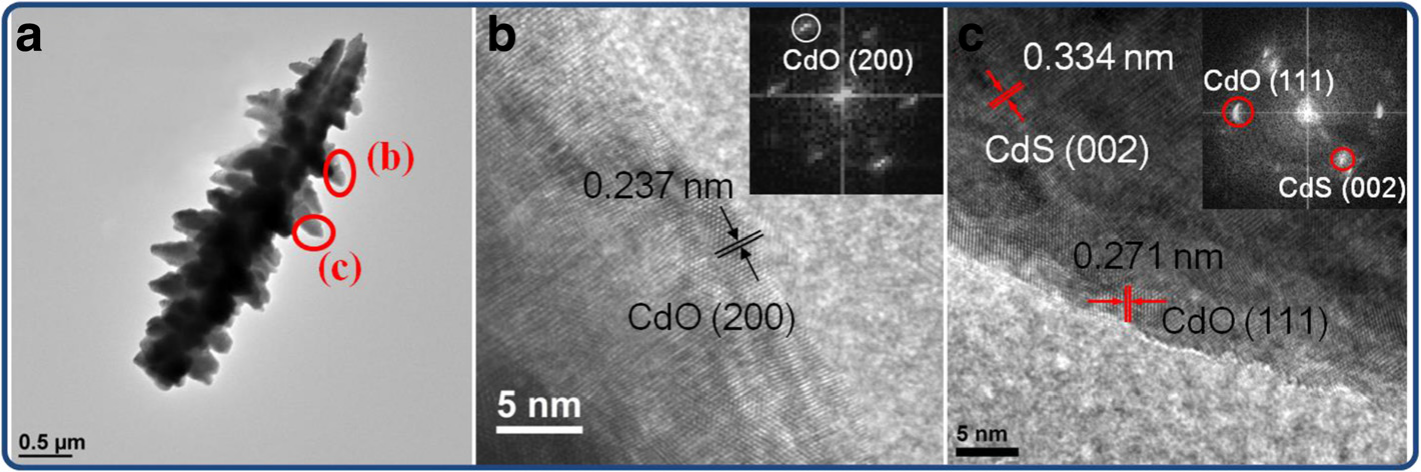
TEM analyses of flower-like CdO@CdS heterostructure. **a** Low-magnification TEM image. **b**, **c** HRTEM images taken from the edges of CdO@CdS heterostructure, and the corresponding FFT patterns are shown in the *insets*

Figure 4a depicts a low-magnification TEM image of the rod-like CdS crystallites, and Fig. 4b shows the selected area electron diffraction (SAED) pattern obtained from the rod-like CdS crystallites in Fig. 4a. The rod-like CdS crystallites had a homogeneous diameter of approximately 10 nm. Moreover, the hexagonal CdS (100), (002), and (101) were indexed on the SAED pattern, and these crystallographic planes are in close agreement with the XRD results. Figure 4c shows the energy-dispersive X-ray spectroscopy (EDS) spectra obtained from the rod-like CdS crystallites, confirming that Cd and S formed the major elemental composition of the sample. Figure 4d, e shows the high-resolution TEM images taken from the local regions (marked with red circles in Fig. 4a) of various rod-like CdS crystallites. Clear and ordered lattice fringes in the CdS crystallites revealed that the as-synthesized rod-like CdS crystallites exhibited a high degree of crystallinity. The atomic lattice fringes had an interval of approximately 0.334 nm, which corresponds to the interatomic distance of hexagonal CdS (002). The corresponding fast Fourier transform (FFT) patterns of the CdS crystallites are shown in the insets of Fig. 4d, e. The FFT patterns show the crystal orientation of the single crystalline CdS herein. These results indicate that each rod-like CdS crystallite is in a single crystalline, and the crystal growth of the rod-like CdS crystallite is along the *c*-axis of the hexagonal phase. Figure 5a–c shows a series of low-magnification TEM images of single and grouped flower-like CdS crystallites. The EDS spectrum showed that the pure flower-like CdS crystallites were formed without any impurities (Fig. 5d). Figure 5e, f shows the HRTEM images obtained from the local regions of various CdS twigs. The lattice fringes with an interval of approximately 0.334 nm corresponded to the lattice constant of hexagonal CdS (002). The clear FFT patterns in the insets of Fig. 5e, f exhibit a high atomic order of the CdS phase in the selected regions. The formation of CdO@CdS heterostructures through the annealing of the flower-like CdS crystallites in ambient air was difficult to identify using the XRD pattern and SEM image because of the trace content of the CdO phase in the heterostructures. The successful synthesis of the CdO@CdS heterostructures was further confirmed by TEM analyses (Fig. 6). As shown in the low-magnification TEM image (Fig. 6a), the morphology of the flower-like CdO@CdS heterostructure had no marked change compared with that of the flower-like CdS crystallite without postthermal annealing. Figure 6b, c shows the HRTEM images obtained from the edges of the heterostructure. Clear and ordered lattice fringes with intervals of approximately 0.237 and 0.271 nm, corresponding to cubic CdO (200) and CdO (111), respectively, were observed (JCPDS no. 005–0640), and the lattice fringes with an interval of approximately 0.334 nm in the internal region matched the spacing distance of hexagonal CdS (002). Moreover, the FFT patterns in the insets of Fig. 6b, c confirm the formation of a crystalline CdO phase in the outer region of the heterostructure when the CdS crystallites underwent postthermal annealing.

**Fig. 7:**
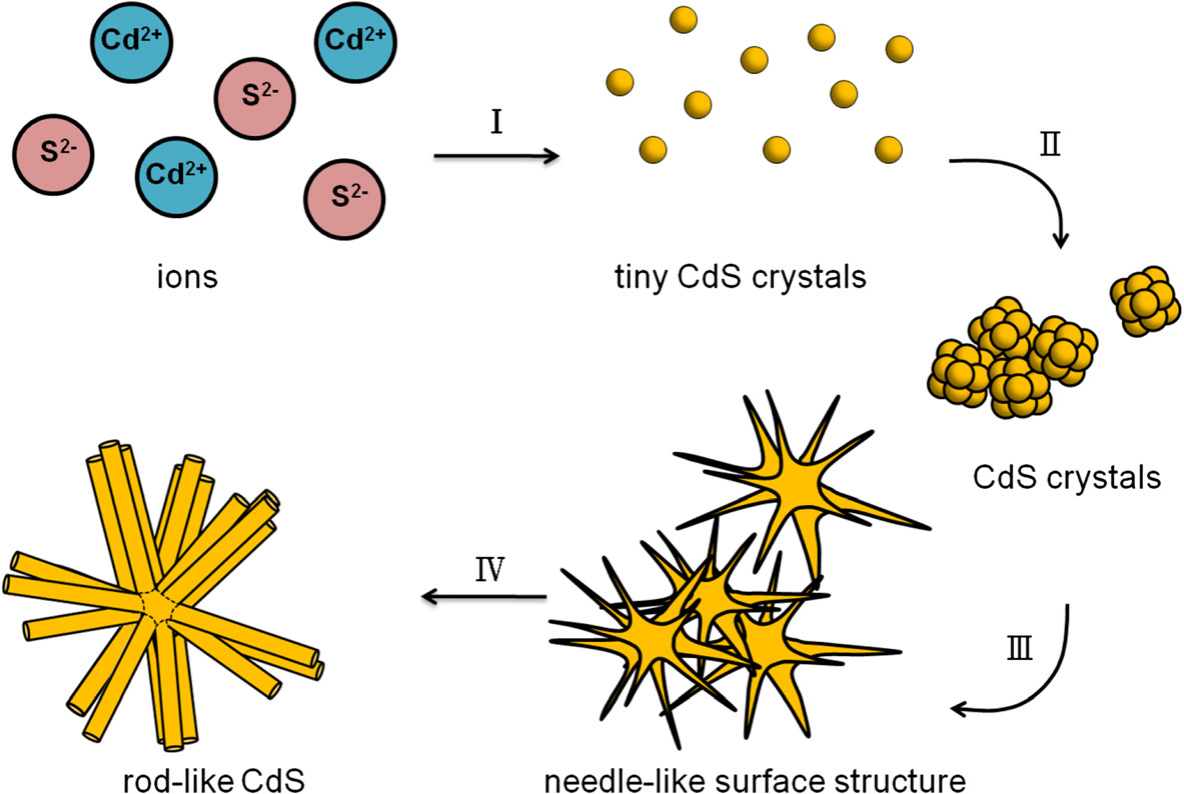
Schematic diagram shows the possible growth mechanism of rod-like CdS structure

**Fig. 8:**
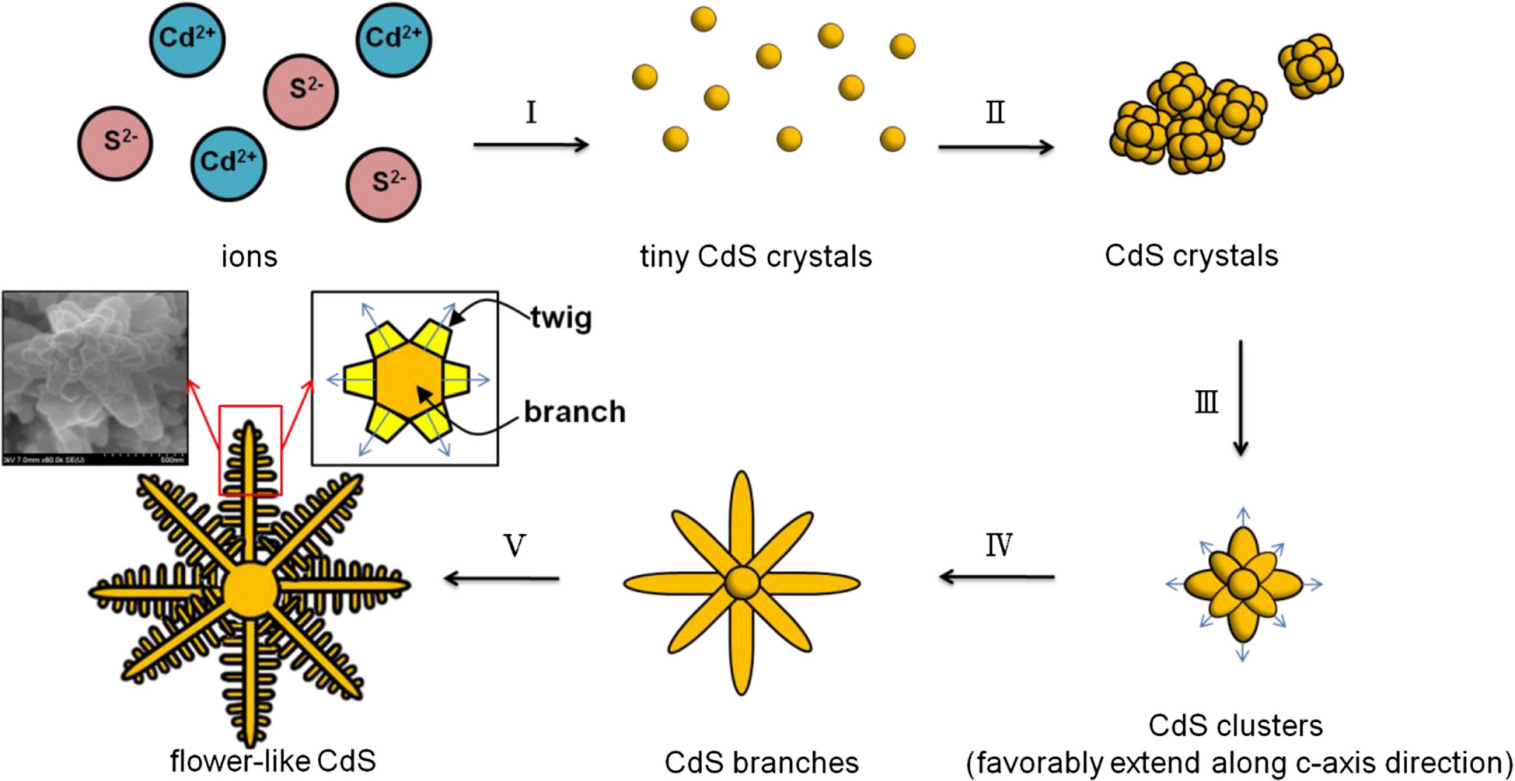
Schematic diagram shows the possible growth mechanism of flower-like CdS structure

The crystal growth mechanism for the rod-like CdS crystallites is schematically shown in Fig. 7. According to the aforementioned structural information, the possible crystal growth mechanism of the rod-like CdS architecture could be divided into four stages. At the hydrothermal reaction stage, alkaline medium S^2−^ ions were generated by the hydrolysis of thiourea, whereas Cd^2+^ ions were generated by the hydrolysis of cadmium nitrate [22]. Upon heating the reaction solution during the initial hydrothermal reaction stage, the complex precursors were decomposed, and S^2−^ ions were gradually increased with the reaction time and then reacted with Cd^2+^ ions to nucleate tiny and dispersed CdS crystals in the reaction solution (the first stage). The as-formed tiny CdS crystals further underwent bonding interaction among the adjacent tiny CdS crystals because of a drop in surface energy. The tiny crystals then began to assemble to form CdS crystals with a sufficient size to be stable exist in the reaction solution in the second stage. When the reaction duration was further increased, these CdS crystals formed large and irregular aggregates. Notably, because of its strong coordination interaction with metal ions, ethylenediamine has been shown to play a crucial role in promoting the growth of hexagonal ZnS crystals along its *c*-axis orientation to form one-dimensional rod-like nanostructures through an aqueous chemical method [23]. It was therefore suggested in the current study that the ethylenediamine surfactant acted as a template-directing agent, continuously inducing the Cd^2+^ and S^2−^ ions in the reaction solution to adsorb with favorable directions onto the as-formed CdS aggregates. The addition of ethylenediamine in the reaction solution might promote the continuous growth of the newly formed CdS crystals along the *c*-axis crystal facet on the CdS aggregates; CdS aggregates with a needle-like surface structure were consequently formed (third stage). At the final stage, because of a relatively fast growth rate of hexagonal CdS along the *c*-axis crystal orientation [24], the rod-like CdS crystals were finally formed on the basis of the continuous growth of the needle-like CdS crystals along the *c*-axis until the reactants were almost depleted during the hydrothermal reaction. Figure 8 shows the growth mechanism of the flower-like CdS crystallites. The first and second stages herein are similar to those of rod-like CdS crystallites. Faceted CdS crystals with a definite size were formed and dispersed on the substrate. These small CdS crystals aggregated to form large CdS clusters with irregular architectures when the hydrothermal reaction duration was further increased (third stage). The distinct extrusion of the CdS crystals grew from the faceted CdS of the clusters and favorably extended along the *c*-axis direction to form large CdS branches (fourth stage). Moreover, the relatively small secondary CdS crystals extended from the side of the initially grown CdS branches and toward specific directions to form CdS twigs. Every twig grew at an angle interval of approximately 60° owing to the six-side facets of the hexagonal structure of CdS. Finally, CdS crystallites with a suitable flower-like architecture were formed on the substrate (fifth stage). A similar flower-like architecture was observed in electrochemically grown ZnO crystals because the crystal growth rate of (001) is faster than that of the crystallographic planes of the six-side facets in the hexagonal wurtzite ZnO [25].

**Fig. 9:**
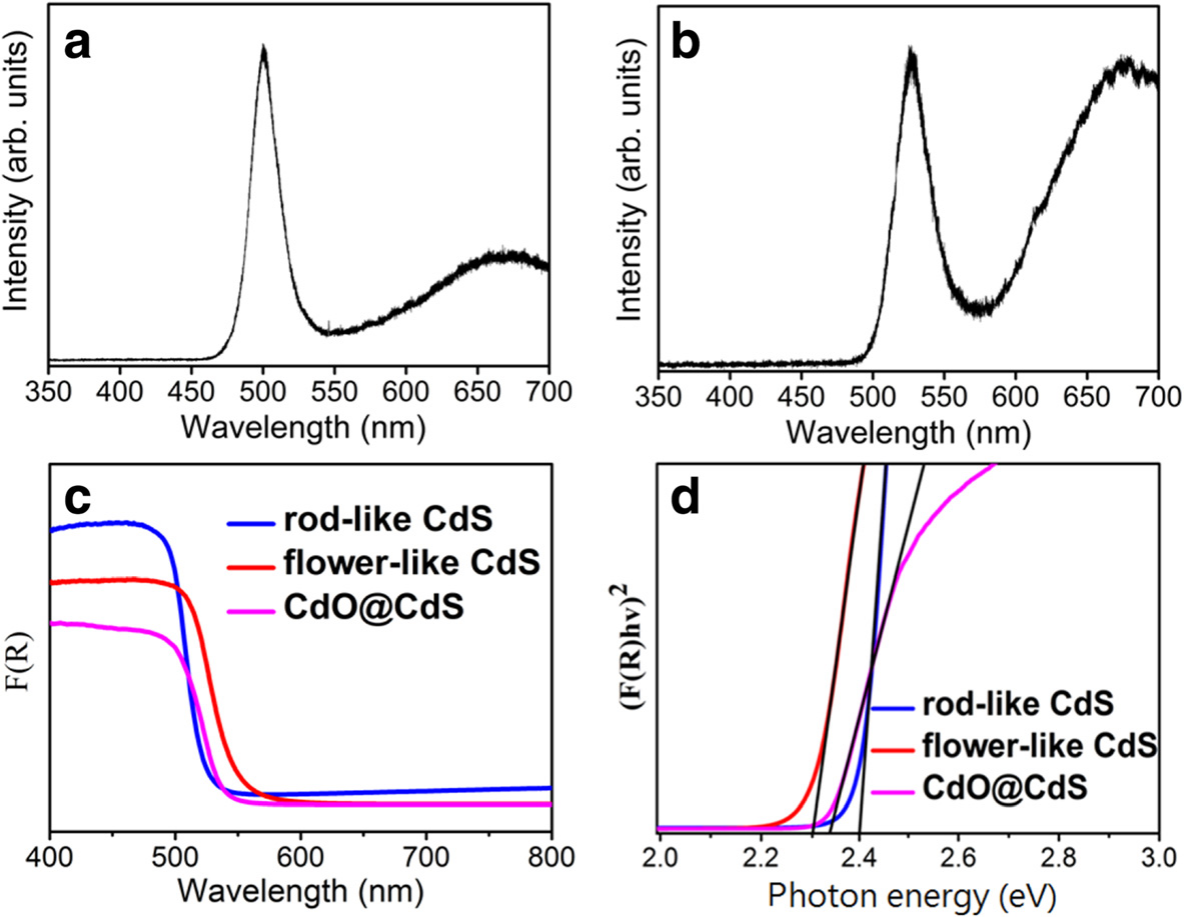
Optical properties of the samples. **a** PL spectrum of rod-like CdS crystallites. **b** PL spectrum of flower-like CdS crystallites. **c** Diffuse reflectance spectra of rod-like CdS, flower-like CdS, and CdO@CdS heterostructure. **d** Tauc plots of rod-like CdS, flower-like CdS, and CdO@CdS heterostructure for evaluating the optical band gap values

Figure 9a, b shows the PL spectra of the rod- and flower-like CdS crystallites. Notably, two emission bands were observed for the rod- and flower-like CdS crystallites. The peaks of emission bands centered at approximately 500 and 527 nm corresponded to the near band-edge emission of the CdS crystallites [26]. Crystal defect-related emission bands of CdS were reported; the emission bands centered at approximately 540–650 nm were attributed to the shallow trap engendered by surface states or the recombination of holes in valence band electrons with deeply trapped S^2−^ vacancies in CdS (V^+^_S_) [27]. On the basis of the aforementioned information, it is reasonable to determine that the peaks of emission bands centered at approximately 665 nm herein originated from the recombination of trapped electrons–holes in some surface states or bulk defects [27]. The flower-like CdS crystallites had a higher intensity ratio of the emission band at 665 nm to the near band-edge emission than did the rod-like CdS crystallites, revealing that the flower-like CdS crystallites are more crystal defective compared with the rod-like CdS crystallites. To understand the optical band gap value of the CdS crystallites with various morphologies, the current study further measured diffuse reflectance spectra of the samples and converted them into absorption coefficient spectra by applying the Kubelka–Munk function [28]. For comparison, the data of the CdS crystallites annealed in the ambient atmosphere are presented in Fig. 9c. As shown in the Kubelka–Munk conversion spectra, the rod-like CdS crystallites, flower-like CdS crystallites, and CdO@CdS heterostructure displayed excellent optical absorption capabilities and spectral intensity variation in the 400–800-nm light wavelength range. The optical band gap values of the samples were estimated according to the theory of Tauc’s formula for direct band gap semiconductors and are presented in Fig. 9d [29]. The optical band gap values of the rod- and flower-like CdS crystallites were approximately 2.4 and 2.3 eV, respectively. The optical band gap value of the CdO@CdS heterostructure was approximately 2.32 eV. No marked change in the optical absorption edge occurred when the flower-like CdS crystallites were thermally annealed; the formation of a trace content of the CdO phase on the CdS surfaces after thermal annealing might explain this observation.

**Fig. 10:**
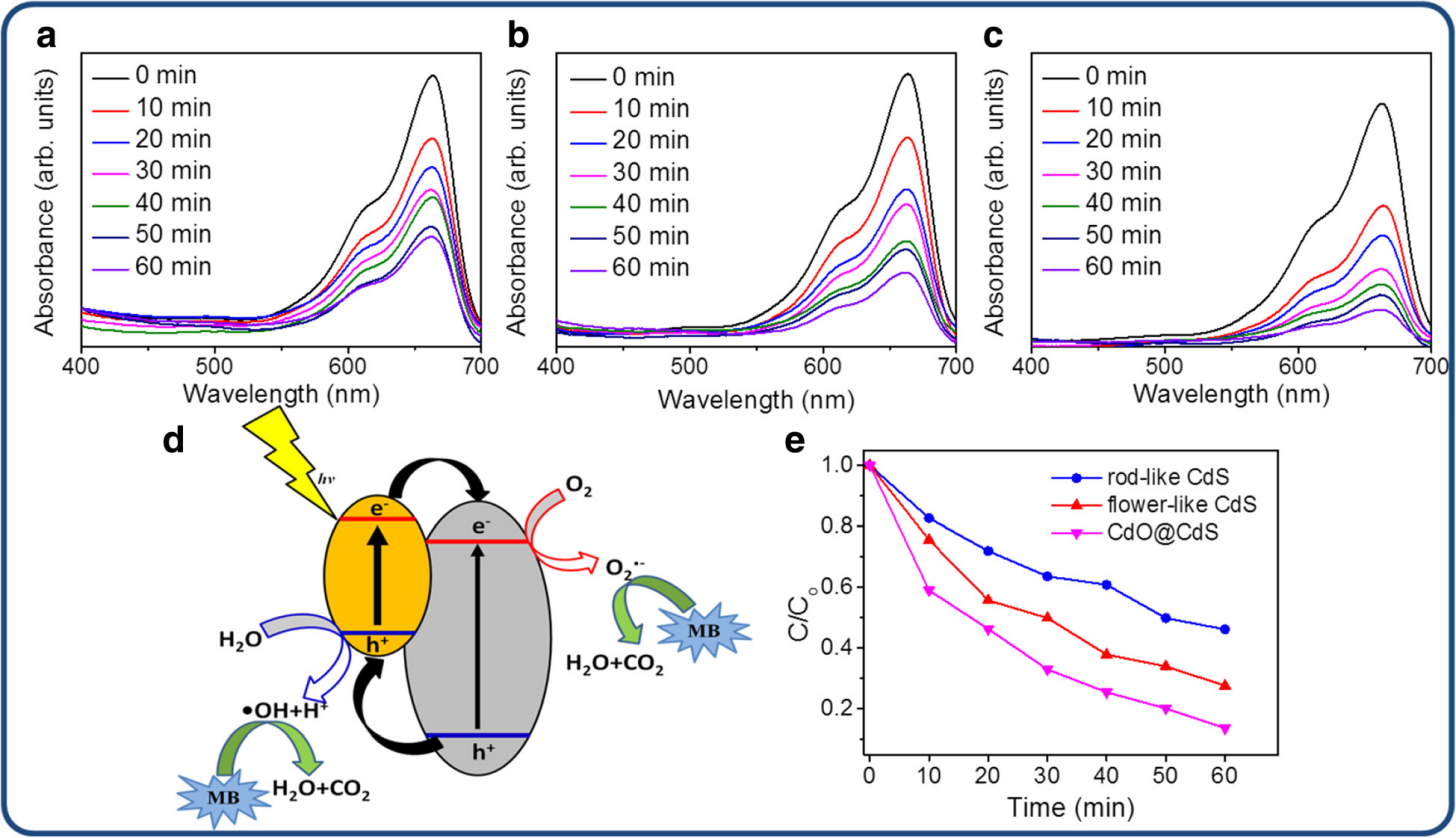
Intensity variation of absorbance spectra of MB solution vs. degradation duration with various samples as photocatalysts under visible light irradiation. **a** Rod-like CdS. **b** Flower-like CdS. **c** CdO@CdS heterostructure. **d** Illustration of photocatalytic reactions of CdS with MB solution. **e** Photodegradation performance of various samples to MB solution

**Fig. 11:**
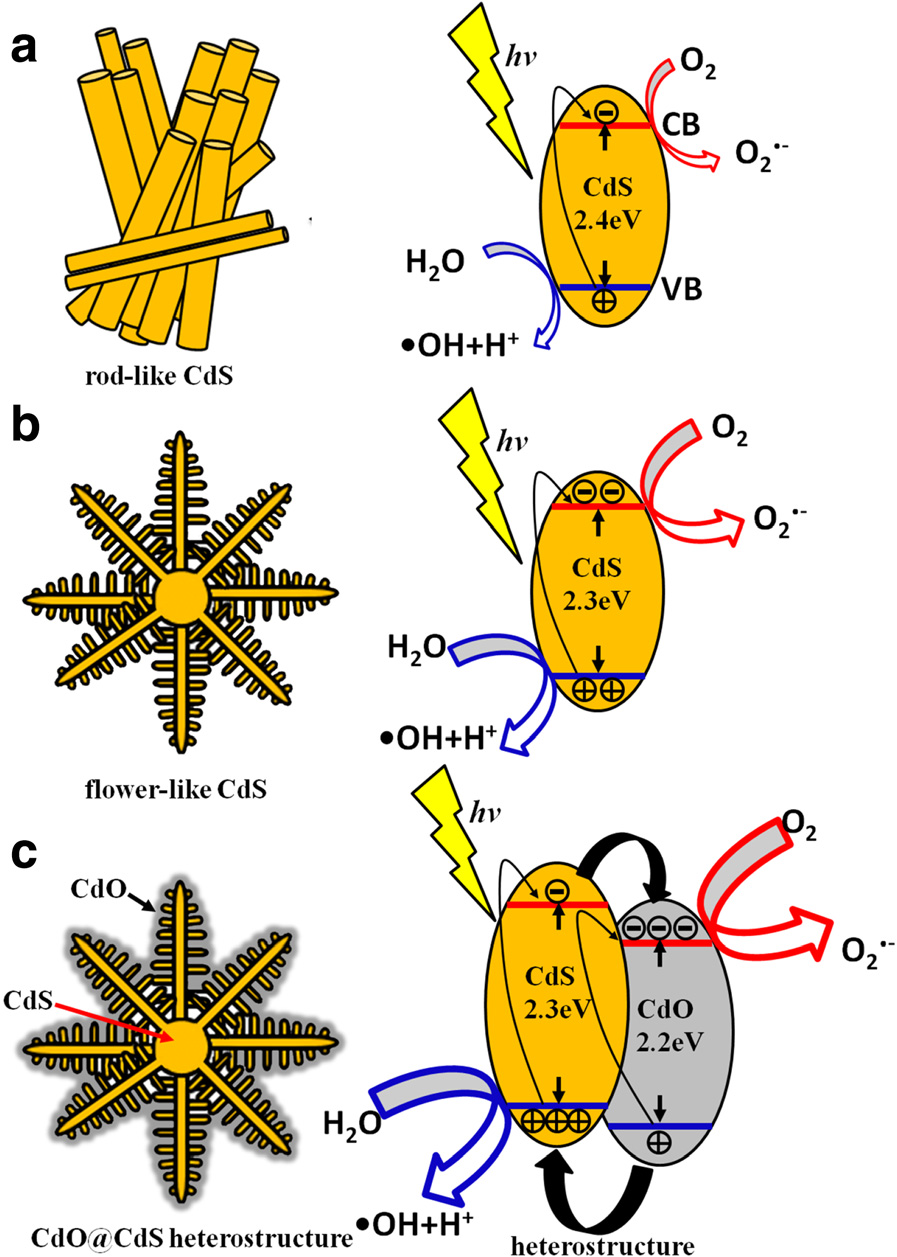
Illustrations of photodegradation performance for various CdS samples. **a** Rod-like CdS. **b** Flower-like CdS. **c** CdO@CdS heterostructure. The size of *blue and red arrows* represents the degree of reaction

Figure 10a–c shows the variations in the intensity of the absorbance spectra of the MB solution containing different CdS samples as catalysts upon exposure to visible light irradiation at various durations. The intensity of the absorbance spectra of the MB solution decreased with the duration of visible light irradiation. This is because during photodegradation, photoexcited electrons or holes in the semiconductor were transferred to the active surface and join in the redox reactions, in which the electrons reduced the dissolved molecular oxygen to produce superoxide radical anions (•O_2_^−^). The unstable •O_2_^−^ further reacted with water rapidly, producing hydroxyl radicals (•OH). Moreover, the holes oxidized H_2_O molecules to yield hydroxyl radicals, and these radicals were strong oxidizing agents and effectively decomposed the MB herein (Fig. 10d) [30]. Figure 10e shows the summarized photodegradation efficiencies of the CdS crystallites with various morphologies and postthermal annealing. Comparatively, the degradation activities followed the following order: CdO@CdS > flower-like CdS > rod-like CdS crystallites. Figure 11a–c illustrates the photodegradation performance of these CdS samples. The sizes of red and blue arrows represent the degree of reaction between the radicals and MB. The photocatalytic activity of the flower-like CdS crystallites was greater than that of the rod-like CdS crystallites; this finding can be attributed to the lower optical band edge of the flower-like CdS crystallites, which broadened the optical absorption range compared with that of the rod-like CdS crystallites. The absorption band edge of the semiconductor photocatalysts shifted to the red region, indicating that the photocatalysts can utilize more solar light, thus enhancing their photocatalytic performance. Several studies have proposed that a similar optical absorption edge shift to a lower energy region improved the photocatalytic ability of semiconductors [31, 32]. Moreover, a study posited that more active sites on the surfaces of catalyst materials are favorable to promote their photoactivated degradation reaction with organic pollutants [33]. Surface defects may serve as charge carrier traps as well as adsorption sites, where the charge transfers to the adsorbed species and prevents electron–hole recombination [34]. In the current study, compared with the rod-like CdS crystallites, a relatively high crystal defect density in the flower-like CdS crystallites, as demonstrated by the PL analysis, was beneficial for improving the efficiency of photodegradation. Notably, the optical band gap of the CdO@CdS heterostructure was not smaller than that of the flower-like CdS crystallites; however, its photodegradation activity was superior to that of the pure CdS crystallites with various morphologies. This observation might be explained by the favorable separation of photogenerated charge carriers engendered by the existence of heterointerfaces in CdO@CdS (Fig. 11c). The ZnO–TiO_2_ heterostructure is proved to manifest superior photocatalytic activity to that of the single constituent compound of ZnO or TiO_2_; the enhanced photocatalytic activity of the heterostructure is primarily attributed to the formation of a heterojunction structure in the interface between ZnO and TiO_2_, which greatly promotes efficient separation of photogenerated electron–hole charge carriers [30]. In the current study, the band alignment at the CdS–CdO interface facilitated the effective transfer of the photogenerated electrons from the conduction band of CdS to the conduction band of CdO. A similar band alignment at the interface of the ZnO–ZnSe heterostructure was reported to play a crucial role in enhancing the photocatalytic activity of the ZnO–ZnSe heterostructure [35]. The band alignment between CdS and CdO might contribute to the spatial separation of electrons and holes, engendering an increase in minority carrier lifetime [36]. Therefore, compared with those of the single CdS, the higher number of charge carriers of the heterostructure could serve the redox reaction during photodegradation. An increased yield of superoxide radical anions and hydroxyl radicals from the photoactivated CdO@CdS heterostructure in the MB solution improved the photodegradation efficiency of MB. Therefore, the photodegradation performance of the flower-like CdS crystallites was enhanced herein by partially oxidizing their surfaces with moderate postthermal annealing.

## Conclusions

In this study, rod- and flower-like CdS crystallites were synthesized using a facile hydrothermal growth method. XRD analyses showed that the rod- and flower-like CdS crystallites have mainly hexagonal phases. The faster crystal growth rate on the *c*-axis than on the other crystallographic planes in the hexagonal CdS phase under the given growth condition might account for the anisotropic growth behavior observed in the rod- and flower-like architectures of the CdS crystallites. The flower-like CdS crystallites exhibited more defective crystal features than did the rod-like CdS crystallites. Moreover, the flower-like CdS crystallites exhibited a broader visible light absorption range than that of the rod-like CdS crystallites. Therefore, the photoactivity of the flower-like CdS crystallites is higher than that of the rod-like CdS crystallites. The postannealing of the flower-like CdS crystallites in ambient air also resulted in the formation of a CdO phase on the surfaces of these crystallites. In this study, the existence of heterointerfaces in the CdO@CdS heterostructure was beneficial for the separation of photogenerated charge carriers, and therefore, the photoactivity of the flower-like CdS crystallites was further improved.
